# Development and initial psychometric evaluation of the LA-Mental Health Scale in working adults

**DOI:** 10.3389/fpsyg.2026.1823291

**Published:** 2026-06-10

**Authors:** María José Rosas-Carmona, Said Jiménez, Fresia Paloma Hernández-Moreno

**Affiliations:** 1 Cuéntame, Clinical Strategy & Research, Mexico City, Mexico; 2 Escuela de Medicina y Ciencias de la Salud, Tecnologico de Monterrey, Mexico City, Mexico; 3 Unidad de Investigación en Medicina Basada en Evidencias, Hospital Infantil de México Federico Gómez, National Institute of Health, Mexico City, Mexico; 4 Escuela de Medicina y Ciencias de la Salud, Tecnologico de Monterrey, Monterrey, Nuevo Leon, Mexico

**Keywords:** anxiety, mental health, scale validation, sleep, stress, well-being, workplace assessment

## Abstract

**Introduction:**

Mental health is increasingly recognized as a critical determinant of both individual well-being and organizational productivity. However, existing assessment tools in Latin American workplace contexts often lack an integrated approach that captures both psychological distress and positive functioning.

**Methods:**

This study describes the development and initial psychometric evaluation of the Latin American Mental Health Scale (LA-MHS), a 30-item multidimensional instrument assessing Anxiety, Stress, Subjective Well-being, and Sleep Problems in working adults. A non-probabilistic sample of 308 workers from multiple Latin American countries completed the scale online. The internal structure was evaluated through theory-driven and data-driven phases, consistent with recommended practices for scale validation.

**Results:**

In the theory-driven phase, confirmatory factor analysis (CFA; WLSMV estimator) supported a four-factor correlated structure with acceptable fit, χ^2^ (395) = 1025.76, *p* < 0.001, CFI = 0.933, TLI = 0.926, RMSEA = 0.072, SRMR = 0.062. Internal consistency was excellent for Anxiety and Stress (α = 0.91, ω = 0.93 for both), acceptable for Well-being (α = 0.74, ω = 0.84), and below recommended thresholds for Sleep Problems (α = 0.68, ω = 0.69). A preliminary brief version (LA-MHS-12) was derived, showing acceptable fit in the same sample, although results should be interpreted cautiously due to potential overfitting. In the data-driven phase, exploratory factor analysis (EFA) in a training subsample identified a three-factor structure (Anxiety, Stress including sleep problems, and Well-being), which was subsequently supported in an independent testing subsample (CFA: CFI = 0.927, TLI = 0.920, RMSEA = 0.083, SRMR = 0.078). Reliability estimates were excellent for Anxiety (α = 0.91, ω = 0.93) and Stress including sleep problems (α = 0.91, ω = 0.93) and good for Well-being (α = 0.80, ω = 0.81). Competing models (higher-order and bifactor) were also examined but did not provide clear advantages in terms of interpretability.

**Discussion:**

Overall, the LA-MHS demonstrates promising initial psychometric properties, particularly for anxiety and stress. However, further refinement of the well-being and sleep dimensions and additional validation in independent samples are required.

## 1 Introduction

Mental health is widely recognized as a fundamental determinant of individual well-being and functioning (World Health Organization, 2014; Keyes, 2002). In occupational settings, mental health problems are consistently associated with reduced job performance, increased absenteeism and presenteeism, higher turnover rates, and substantial economic and human costs (Demerouti et al., 2001; Hassard et al., 2018; Sverke et al., 2002). Among the most prevalent psychological conditions, anxiety and stress play a central role in shaping mental health outcomes (Kessler et al., 2005; American Psychiatric Association, 2013; Beck and Clark, 1997). Both have been linked to functional impairment, reduced productivity, and increased risk for mental and physical disorders. Importantly, these conditions frequently co-occur with sleep disturbances and diminished subjective well-being, forming a highly prevalent and interconnected psychological risk profile. In the absence of timely prevention and intervention, this profile may persist or worsen over time (Harvey, 2008; Buysse, 2014; Ganster and Rosen, 2013).

Empirical evidence highlights reciprocal relationships among these dimensions. Elevated stress is associated with increased anxiety and poorer sleep quality, whereas sleep disturbances can heighten emotional reactivity and impair affect regulation, ultimately undermining well-being (Alvaro et al., 2013; Baglioni et al., 2010; Baglioni et al., 2011). These interdependencies underscore the importance of assessing mental health in an integrated manner, rather than through isolated constructs. Despite this, mental health assessment in applied settings is often conducted using unidimensional instruments, each targeting a single domain. Widely used measures such as the Beck Anxiety Inventory (BAI; Beck et al., 1988), the State–Trait Anxiety Inventory (Spielberger et al., 1983), the Pittsburgh Sleep Quality Index (PSQI; Buysse et al., 1989), and subjective well-being scales (e.g., EBS-20, EBS-8) provide robust assessments of individual constructs. However, their use in workplace settings is often limited by length, lack of integration, and limited cultural adaptation to Latin American contexts (Table 1). As a result, organizations frequently lack brief, multidimensional tools capable of capturing both psychological distress and positive functioning within a single, actionable framework.

**Table 1:** Comparison of commonly used mental health instruments and the LA-MHS.

Scale	Evaluated dimensions	Application in occupational settings	Validation in LA population	Integrated assessment (anxiety, stress, well-being and sleep)
STAI (Spielberger et al., 1983)	Anxiety (state and trait)	Limited use (primarily clinical and research settings)	Yes	No
BAI (Beck et al., 1988)	Anxiety	Limited use (clinical focus)	Yes	No
PSQI (Buysse et al., 1989.)	Sleep quality	Occasional	Yes (Spanish-language adaptations)	No
Subjective Well-being Scale EBS-20	Subjective well-being	Limited	Yes	No
NOM-035 (STPS)	Psychosocial risk factors	Yes (mandatory in Mexico)	Yes	No
Proposed scale - LA-Mental Health Scale (LA-MHS)	Anxiety, stress, sleep and well-being	Yes	In process	Yes

In Mexican organizations, assessment efforts have focused primarily on psychosocial risk factors rather than on individual psychological states. For example, the Mexican Official Standard, NOM-035-STPS-2018, outlines procedures for identifying workplace psychosocial risks. However, this standard is not intended as a mental health diagnostic tool or a comprehensive measure of individual functioning, as its main focus is on organizational conditions rather than subjective internal experiences. Furthermore, many existing instruments used in Latin American countries were developed in English-speaking contexts, raising concerns about cultural and semantic equivalence for the target population (Hambleton et al., 2005; de Van Vijver and Leung, 2011).

In this context, there is a clear need to develop a brief instrument, consistent with established guidelines in psychological assessment (American Educational Research Association, American Psychological Association, National Council on Measurement in Education, 2014; DeVellis, 2017). This instrument should be designed for screening and monitoring rather than diagnosis, and capable of capturing both psychological distress (anxiety, stress, and sleep problems) and positive functioning (well-being) within a single assessment. Given the dynamic and context-dependent nature of mental health, scores should be interpreted as reflecting an individual’s current psychological state, with potential utility for early identification, prevention, and monitoring over time.

From a theoretical perspective, integrating anxiety, stress, well-being, and sleep within a single instrument aligns with contemporary dimensional models of mental health. The two-continua model posits that well-being and psychological distress are related but partially independent constructs, such that the absence of symptoms does not necessarily imply high well-being (Keyes, 2005; Westerhof and Keyes, 2010; Ryff, 1989; Ryff and Singer, 1998). This perspective is particularly relevant in organizational and preventive contexts, where the goal is not diagnosis but the identification of risk and protective profiles. Similarly, transdiagnostic frameworks emphasize shared underlying processes across emotional conditions, providing further rationale for assessing these dimensions jointly. Together, these perspectives support the conceptualization of mental health as a multidimensional construct and provide the theoretical basis for the instrument developed in the present study. The Latin American Mental Health Scale (LA-MHS) was designed to assess four correlated dimensions representing both negative and positive aspects of mental health: Anxiety, Stress, Subjective Well-being, and Sleep Problems.

Anxiety was conceptualized as an emotional response involving excessive worry, hypervigilance, and physiological activation in anticipation of future threat (Barlow, 2002). Psychological stress, in contrast, reflected a transactional process in which perceived demands exceed coping resources (Lazarus and Folkman, 1984). In occupational contexts, the Job Demands–Resources model highlights how sustained imbalance between demands and resources leads to exhaustion and reduced well-being (Demerouti et al., 2001; Bakker and Demerouti, 2017). Subjective well-being encompasses individuals’ cognitive and affective evaluations of their lives (Diener et al., 1999; Diener, et al., 1985), while sleep is a fundamental biological process essential for restoration and emotional regulation (Walker, 2017), with well-established bidirectional links to stress and anxiety (Harvey, 2008; Baglioni et al., 2010; Baglioni et al., 2011).

The aim of this study was to develop and psychometrically evaluate the Latin American Mental Health Scale (LA-MHS). The evaluation was conducted in three complementary phases. The first phase was theory-driven and guided by the initial item development. In this phase, a hypothesized four-factor internal structure was directly evaluated. Specifically, it was expected that: (1) the instrument would exhibit a correlated four-factor structure; (2) anxiety, stress, and sleep problems would be positively associated; and (3) well-being would be negatively associated with distress-related dimensions. An additional objective of this phase was to link the proposed theoretical structure with empirical findings to inform the development of a brief version of the LA-MHS.

The second phase was data-driven and aimed to explore alternative factorial structures in one subset of the data and subsequently evaluate their fit in an independent subset. This phase was considered exploratory; therefore, no specific hypotheses were proposed. Finally, the third phase aimed to estimate and compare competing latent structures, including the correlated four-factor model, a higher-order model, and a bifactor model. No specific hypotheses were formulated for this phase.

## 2 Materials and methods

### 2.1 Study design

A cross-sectional psychometric validation study was conducted to develop and psychometrically analyze the LA-MHS in working adults. The development of this instrument was motivated by the need for a brief, reliable, and culturally appropriate tool for screening key mental health variables in workplace contexts from a preventive—rather than diagnostic—perspective.

### 2.2 Participants

Participants were recruited using a non-probabilistic, intentional convenience sampling strategy. A total of 308 valid responses were obtained. Data collection was conducted through three recruitment channels. First, an invitation to participate was disseminated via LinkedIn through a public post that included a link to the scale and was directed to Cuéntame’s professional network. Second, a referral-based strategy (word-of-mouth sampling) was implemented, whereby collaborators shared the assessment link within their professional networks. Third, the scale was distributed through databases of companies that voluntarily agreed to participate. Due to confidentiality agreements, the names of these organizations are not disclosed. Participation was voluntary, anonymous, and without financial compensation. Before accessing the questionnaire, participants reviewed and electronically accepted an informed consent form explaining the study objectives, data use, and confidentiality safeguards. Table 2 presents the sociodemographic characteristics of the sample (*N* = 308). The sample was primarily composed of participants from Mexico (84.1%), with additional representation from other countries including Colombia (8.1%), Peru (2.3%), Ecuador (1.3%), and others representing the remaining 4.2%. Participants were predominantly between 18 and 35 years old, and most worked in education, social sciences, or creative sectors.

**Table 2:** Sociodemographic characteristics of the study participants (*N* = 308).

Variable	Total (*n* = 308)
Gender	
Women	190 (61.7%)
Men	116 (37.7%)
Prefer not to say	2 (0.6%)
Country of residence	
Mexico	259 (84.1%)
Colombia	25 (8.1%)
Perú	7 (2.3%)
Ecuador	4 (1.3%)
Bolivia	3 (1.0%)
Argentina	2 (0.6%)
El Salvador	2 (0.6%)
Costa Rica	2 (0.6%)
Guatemala	1 (0.3%)
Venezuela	1 (0.3%)
United States	1 (0.3%)
Panamá	1 (0.3%)
Age group (years)	
18–25	70 (22.7%)
26–30	76 (24.7%)
31–35	48 (15.6%)
36–40	38 (12.3%)
41–45	33 (10.7%)
46–50	10 (3.2%)
51–55	9 (2.9%)
56 o más	24 (7.8%)
Functional area	
Education/Social Sciences/Creative	201 (65.3%)
Technology	38 (12.3%)
Administration and Finance	30 (9.7%)
Health and Well-Being	23 (7.5%)
Operations and Production	16 (5.2%)

Values are reported as frequency (n) and percentage (%). Percentages are based on the total sample size. Work areas were initially collected through open-ended responses and subsequently categorized into broader functional areas for descriptive purposes.

The sample size was determined based on commonly used guidelines in psychometric research, which recommend a minimum ratio of participants per item (e.g., 10:1) in factor analytic studies (Worthington and Whittaker, 2006). A criterion of at least 10 participants per item was considered appropriate for the present study. Given that the full version of the scale includes 30 items, the final sample of 308 participants met this requirement.

Given the non-probabilistic and convenience-based nature of the sampling strategy, the sample may not be fully representative of the broader working population. This approach may introduce selection biases (e.g., overrepresentation of certain sectors or individuals with higher digital access), which should be considered when interpreting the generalizability of the findings. The sample showed a higher representation of women, younger participants, and individuals working in specific sectors, which should be considered when interpreting the scope of findings.

### 2.3 Scale development

Item generation involved an iterative process in which the research team reviewed existing validated instruments such as the Generalized Anxiety Disorder–7 (GAD-7; Spitzer et al., 2006), the Perceived Stress Scale (Cohen et al., 1983), and theoretical models (transdiagnostic frameworks, the Job Demands–Resources (JD–R) model) to identify core constructs and commonly used item formulations. Items were adapted and reworded to ensure clarity, brevity, and cultural relevance for Latin American workplace contexts. Draft items were internally reviewed by the research team and external mentors, and refined through some iterations (including a pilot with 54 participants), to reduce redundancy and improve conceptual alignment across dimensions.

The well-being dimension was grounded in the two-continua model of mental health, which conceptualizes well-being as related to but partially independent from psychological distress. Item construction was aligned with established measures such as the WHO-5 Well-Being Index and the Mental Health Continuum–Short Form (Keyes, 2005), incorporating indicators of positive functioning, including life satisfaction, enjoyment, perceived purpose, everyday self-efficacy, and social connectedness. The sleep dimension was included as a cross-cutting indicator of mental health due to its documented bidirectional associations with anxiety, stress, and well-being. Item development was informed by the conceptual framework of the Pittsburgh Sleep Quality Index (PSQI), focusing on difficulties in sleep initiation and maintenance, as well as daytime functional consequences. Together, these dimensions informed the development of a multidimensional screening instrument aimed at identifying psychological risk and resource profiles in workplace contexts.

The final version of the Latin American Mental Health Scale (LA-MHS) for the workplace consists of 30 items distributed across four dimensions: Anxiety (10 items), Stress (10 items), Well-being (7 items), and Sleep Problems (3 items). Items are phrased as statements reflecting subjective experiences during the past four weeks and are rated on a five-point Likert-type scale ranging from 1 (strongly disagree) to 5 (strongly agree). A brief version (LA-MHS-12) was derived by selecting the three items per dimension based on their standardized factor loadings, while also considering conceptual relevance and content coverage within each construct. This approach aimed to retain the core conceptual meaning of each dimension while maximizing parsimony. However, this data-driven approach may carry risks such as overfitting or reduced content breadth, and therefore the brief version should be further validated in independent samples.

### 2.4 Procedure

The scale was administered online through Cuéntame’s digital platform, which provides mental health assessment and preventive services in organizational contexts in Mexico and other Latin American countries. Participation was voluntary, self-administered, and uncompensated. Prior to accessing the questionnaire, participants reviewed and provided electronic informed consent within the survey platform (Online form: http://app.sicuentame.com/escala_proyecto). The consent process ensured anonymity and confidentiality of responses. The estimated completion time was approximately 5–6 min.

Inclusion criteria required participants to be adults currently engaged in paid work. Responses were screened for completeness (we did not have any cases with incomplete answers).

### 2.5 Data analysis

The psychometric evaluation was conducted in three phases, consistent with the study aims. The first phase (theory-driven) involved fitting a confirmatory factor analysis (CFA) using the full sample of 308 participants who completed the LA-MHS. A correlated four-factor structure was specified, in which each item loaded exclusively on its corresponding latent factor: anxiety (ANX, items 1–10), stress (STR, items 11–20), well-being (WB, items 21–27), and sleep problems (SLP, items 28–30). Based on this model, the three items with the highest factor loadings within each dimension were selected to construct a brief version of the instrument (LA-MHS-12). The second phase (data-driven) implemented a cross-validation procedure. The sample was randomly split into two equal subsamples: a training sample (n = 154), used to conduct an exploratory factor analysis (EFA), and a test sample (n = 154), used to evaluate the factor structure identified in the training sample via CFA. The third phase involved the estimation and comparison of three competing models using the full sample. The baseline model corresponded to the theory-driven correlated four-factor structure. A second-order model was then specified, in which the correlations among the four first-order factors were explained by a higher-order general factor (G). Finally, a bifactor model was estimated, including a general factor accounting for the common variance across all items, alongside four orthogonal group factors corresponding to the theoretically defined dimensions (ANX, STR, WB, and SLP), which captured residual variance not explained by the general factor.

For the EFA, the number of factors to extract was determined using parallel analysis based on a polychoric correlation matrix, given the ordinal nature of the data. Factor extraction was conducted using the minimum residual (MinRes) method, as implemented in the *psych* package in R (Revelle, 2025). Parallel analysis suggested a three-factor solution; therefore, an EFA with three correlated factors was estimated using oblimin rotation, the polychoric correlation matrix, and the MinRes extraction method. Given the ordinal nature of the items (5-point Likert scale), all CFA’s were conducted using the Weighted Least Squares Mean and Variance adjusted (WLSMV) estimator. Unlike Maximum Likelihood, WLSMV does not require multivariate normality of observed variables and instead utilizes polychoric correlations based on the assumption of underlying bivariate normality. Analyses were conducted in R using the *lavaan* package (Yves Rosseel, 2012). For model identification, the factor loading of the first item of each latent variable was fixed to 1 (Brown, 2015). In the second-order model, the loading of the G factor on the first-order factor stress was also fixed to 1. Factor correlations were freely estimated in all models, except in the bifactor model (which assumes orthogonality) and in the second-order model (where associations among first-order factors are accounted for by the higher-order factor). Given the five response categories, four thresholds (k − 1) were estimated for each item. Internal consistency reliability was evaluated using ordinal alpha and omega, appropriate for ordinal response categories.

Prior to analysis, item distributions were screened for sufficient category frequencies and potential outliers. Local independence was assessed via modification indices, with theoretically redundant item pairs addressed through specified residual correlations to improve model parsimony and fit. Convergent and discriminant validity were assessed using Average Variance Extracted (AVE) and the Fornell-Larcker criterion (Fornell and Larcker, 1981). Model fit for the CFA models was evaluated using multiple indices: chi-square (χ^2^), Comparative Fit Index (CFI), Tucker–Lewis Index (TLI), Root Mean Square Error of Approximation (RMSEA), and Standardized Root Mean Square Residual (SRMR). The following criteria were used to indicate acceptable fit: CFI and TLI ≥ 0.90, RMSEA ≤ 0.06, and SRMR ≤ 0.06 (Hu and Bentler, 1999). The chi-square statistic was interpreted cautiously due to its sensitivity to sample size. For the bifactor model, Explained Common Variance (ECV) was additionally computed to quantify the proportion of common variance attributable to the general factor (Dueber and Toland, 2023). Values of ECV > 0.70 were interpreted as indicative of essential unidimensionality (Rodriguez et al., 2016).

## 3 Results

### 3.1 Phase 1 theory-driven approach: *a priori* four-factor correlated structure

This phase evaluated the a priori hypothesized four-factor correlated structure using confirmatory factor analysis (CFA) for the full LA-MHS (30 ordinal items, WLSMV estimator; n = 308). Initial model fit indices indicated acceptable but suboptimal fit, χ^2^ (scaled) = 1126.83, df = 399, *p* < 0.001, CFI = 0.923, TLI = 0.916, RMSEA = 0.077, SRMR = 0.067. Although incremental fit indices (CFI and TLI) met conventional thresholds (≥ 0.90), RMSEA and SRMR suggested moderate model misfit. Inspection of modification indices indicated local dependence among several items within the Well-being factor: Item 24 (“I have easily experienced moments of joy or genuine enjoyment”) with Item 23 (“I have felt satisfied with my personal and work life”), Item 24 with Item 26 (“I have felt valued by the people around me”), Item 24 with Item 21 (“It has been easy for me to feel at peace with myself”), and Item 23 with Item 22 (“I have felt motivated by clear personal goals”). Given the conceptual and semantic similarity among these items, four within-factor residual correlations were specified. No cross-factor residual correlations were included. The respecified model demonstrated improved fit, χ^2^ (scaled) = 1025.76, df = 395, *p* < 0.001, CFI = 0.933, TLI = 0.926, RMSEA = 0.072, SRMR = 0.062. These results indicate an improvement in both incremental and residual-based fit indices, initially supporting the adequacy of the proposed four-factor structure, while also suggesting the need for further external validation.

#### 3.1.1 CFA parameters for the full LA-MHS

Standardized factor loadings in the initial CFA model were positive and statistically significant, ranging from 0.51 to 0.84 (Table 3). The highest-loading item for the Anxiety factor was Item 6 (“I have felt nervous without an apparent reason”), whereas for Stress it was Item 15 (“I have felt emotionally exhausted”). Within the Well-being factor, Item 23 (“I have felt satisfied with my personal and work life”) showed the highest loading, and for Sleep Problems, Item 30 (“During the day, I feel sleepy or lacking energy despite having slept”) had the strongest loading. Interfactor correlations were consistent with hypotheses 2 and 3, showing positive associations among Anxiety, Stress, and Sleep Problems, and negative associations between these dimensions and Well-being. Particularly strong correlations were observed between Anxiety and Stress (r = 0.84, SE = 0.02, *p* < 0.001) and between Stress and Sleep Problems (r = 0.80, SE = 0.03, *p* < 0.001). Parameter estimates from the respecified model, including the four within-factor residual correlations, were highly similar to those obtained from the initial model without residual correlations, initially supporting the proposed four-factor structure. Minor differences were primarily observed within the Well-being factor. Detailed parameter estimates for both models are presented in Table 3.

**Table 3:** Standardized parameters of the confirmatory factor model of the full LA-MHS.

Factor	Parameter	Item/Factor	Model 1		Model 2	
			Est	SE	Est	SE
ANX	→	1. I have felt constantly worried, even without a clear reason.	0.66	0.04	0.66	0.04
		2. I have had difficulty controlling fearful or anticipatory thoughts.	0.75	0.03	0.75	0.03
		3. I have felt restless or unable to relax.	0.8	0.02	0.8	0.02
		4. I have experienced physical anxiety symptoms (e.g., muscle tension, trembling, palpitations).	0.61	0.04	0.61	0.04
		5. I have felt overwhelmed by small problems.	0.77	0.02	0.77	0.02
		6. I have felt nervous without an apparent reason.	0.83	0.02	0.83	0.02
		7. I worry about things others consider minor or unimportant.	0.75	0.02	0.75	0.02
		8. I have avoided situations due to fear of feeling anxious.	0.76	0.03	0.75	0.03
		9. I have felt that something bad might happen without a clear reason.	0.76	0.02	0.76	0.02
		10. I have found myself constantly anticipating negative scenarios.	0.76	0.03	0.76	0.03
STR	→	11. I have felt under pressure most of the time.	0.79	0.02	0.79	0.02
		12. I have felt unable to control important events in my life	0.75	0.03	0.75	0.03
		13. I have had difficulty concentrating due to stress.	0.75	0.03	0.75	0.03
		14. My sleep or appetite has changed because of stress.	0.79	0.02	0.79	0.02
		15. I have felt emotionally exhausted.	0.84	0.02	0.84	0.02
		16. I have felt easily irritated or frustrated.	0.81	0.02	0.81	0.02
		17. I have felt overwhelmed by too many responsibilities and too little time.	0.66	0.03	0.66	0.03
		18. I have found it difficult to mentally disconnect from work tasks.	0.69	0.03	0.69	0.03
		19. I have felt that my problems accumulate faster than I can solve them.	0.81	0.02	0.81	0.02
		20. I have experienced physical pain (e.g., headaches, stomachaches) related to stress.	0.72	0.03	0.72	0.03
WB	→	21. It has been easy for me to feel at peace with myself.	0.57	0.05	0.6	0.05
		22. I have felt motivated by clear personal goals.	0.54	0.05	0.49	0.05
		23. I have felt satisfied with my personal and work life.	0.71	0.04	0.7	0.05
		24. I have easily experienced moments of joy or genuine enjoyment.	0.51	0.07	0.64	0.09
		25. I have felt capable of managing daily challenges.	0.64	0.05	0.63	0.05
		26. I have felt valued by the people around me.	0.63	0.04	0.66	0.04
		27. I have felt that my daily actions align with what matters to me.	0.7	0.04	0.69	0.04
SLP	→	28. I have had difficulty falling asleep.	0.65	0.04	0.65	0.04
		29. I wake up during the night or too early and cannot fall back asleep.	0.66	0.04	0.66	0.04
		30. During the day, I feel sleepy or low in energy despite having slept.	0.75	0.04	0.75	0.04
ANX	↔	STR	0.84	0.02	0.84	0.02
ANX	↔	WB	−0.47	0.05	−0.46	0.05
ANX	↔	SLP	0.69	0.04	0.69	0.04
STR	↔	WB	−0.47	0.05	−0.46	0.05
STR	↔	SLP	0.8	0.03	0.8	0.03
WB	↔	SLP	−0.43	0.06	−0.43	0.06

Model 1 = CFA model with four correlated factors, Model 2 = CFA model with four correlated factors and four within-factor residual correlations, ANX = Anxiety, STR = Stress, WB = Well-being, SLP = Sleep Problems, Est = standardized parameter estimate, SE = standard error. One-headed arrows (→) are standardized factor loadings, and double-headed arrows (↔) represent correlations.

#### 3.1.2 Reliability of the full LA-MHS

Internal consistency was evaluated separately for each factor using ordinal alpha and omega coefficients (Table 4). Anxiety and Stress demonstrated excellent internal consistency across both indices. Well-being showed acceptable reliability according to ordinal alpha and good reliability based on omega. In contrast, the Sleep Problems factor yielded reliability estimates below the commonly accepted threshold (>0.70). While its inclusion contributes to the overall scale, scores for this dimension should be interpreted with caution when considered independently, and separate use is therefore not recommended without further investigation.

**Table 4:** Ordinal alpha and omega internal consistency indices, average variance extracted, and factor correlations for the LA-MHS subdimensions and the brief LA-MHS-12 version.

Version	Factor	α	ω	Factor correlations				
				AVE	ANX	STR	WB	SLP
LA-MHS	ANX	0.91	0.93	0.56	(0.75)			
	STR	0.91	0.93	0.58	0.84	(0.76)		
	WB	0.74	0.84	0.38	−0.47	−0.47	(0.62)	
	SLP	0.68	0.69	0.48	0.30	0.80	−0.43	(0.69)
LA-MHS-12	ANX	0.82	0.83	0.67	(0.82)			
	STR	0.81	0.82	0.66	0.87	(0.81)		
	WB	0.70	0.72	0.51	−0.40	−0.45	(0.71)	
	SLP	0.68	0.69	0.48	0.67	0.75	−0.40	(0.69)

α = ordinal alpha, ω = omega coefficient or Composite Reliability (CR), AVE = Average Variance Extracted, ANX = Anxiety factor, STR = Stress factor, WB = Well-being factor, SLP = Sleep problems. Diagonals in parentheses represent the square root of the AVE. Off-diagonals are factor correlations.

#### 3.1.3 Brief version: LA-MHS-12

Within Phase 1, and as an exploratory step, results from the CFA of the hypothesized four-factor model were used to inform the development of a brief version of the LA-MHS. The three items with the highest factor loadings were selected for the Anxiety, Stress, and Well-being dimensions, while all three available items from the Sleep Problems factor were retained, resulting in the LA-MHS-12. Although proper validation of the LA-MHS-12 requires an independent sample, its internal structure and reliability were preliminarily evaluated in the present dataset using a four-factor CFA model and internal consistency indices (ordinal alpha and omega). Given that this analysis was conducted within the same sample, model fit and parameter estimates may be inflated, and therefore should not be considered definitive. For the LA-MHS-12 (12 items; four correlated factors), global fit indices indicated strong model fit, χ^2^ (scaled) = 145.13, df = 48, *p* < 0.001, CFI = 0.971, TLI = 0.959, RMSEA = 0.081, SRMR = 0.044. Although RMSEA slightly exceeded conventional thresholds (0.081), this index is known to overestimate model misfit in models with low degrees of freedom and when using WLSMV estimation (Shi et al., 2019). Importantly, incremental (CFI, TLI) and residual-based (SRMR) indices indicated, as expected, excellent fit. Taken together, these findings provide initial support for the internal structure of the brief version, although further external validation is warranted.

**Figure 1:**
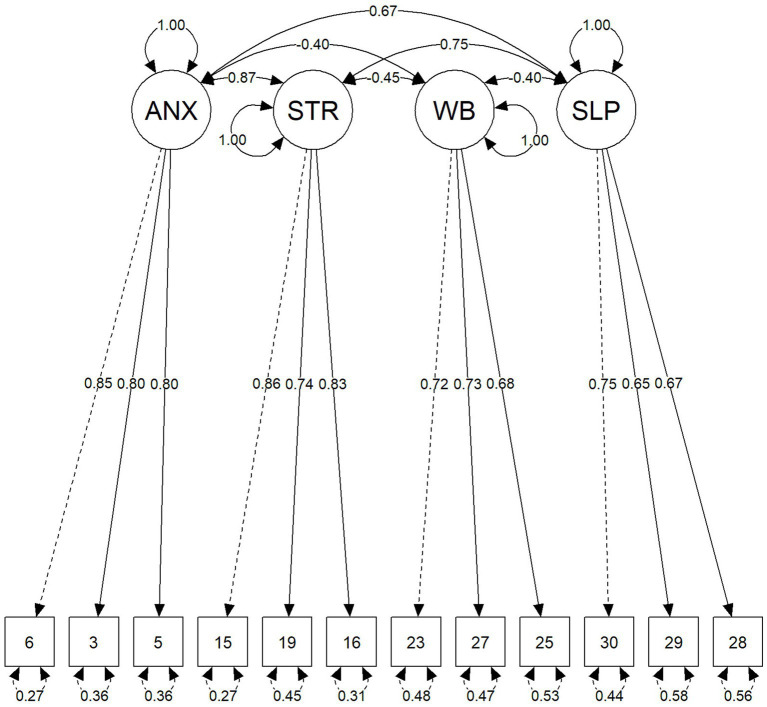
Path diagram of the confirmatory factor model of the brief LA-MHS-12 version. Circles represent the four dimensions assessed by the questionnaire, while rectangles represent the items measuring each dimension. Unidirectional arrows indicate standardized factor loadings, and bidirectional arrows indicate standardized variances or covariances.

Factor loadings in the brief version were positive, statistically significant, and generally stronger than those observed in the full version (Table 5; Figure 1). The correlations found in the LA-MHS-12 closely mirrored the pattern observed in the full version (Tables 3, 5). In terms of reliability, Anxiety and Stress demonstrated good reliability across both ordinal alpha and omega, Well-being showed acceptable reliability, and Sleep Problems again fell below recommended thresholds (Table 4). Overall, these findings suggest that the Anxiety, Stress, and Well-being subscales of the LA-MHS-12 provide reliable scores, whereas additional refinement may be needed for the Sleep Problems dimension before its use in screening and monitoring applications.

**Table 5:** Standardized parameters of the confirmatory factor model of the brief LA-MHS-12 version.

Factor	Parameter	Item/Factor	Est	SE	CI 95%	
					LL	UP
ANX	→	6	0.85	0.02	0.81	0.89
		3	0.8	0.03	0.74	0.85
		5	0.8	0.02	0.75	0.85
STR	→	15	0.86	0.02	0.82	0.89
		19	0.74	0.03	0.68	0.8
		16	0.83	0.02	0.79	0.88
WB	→	23	0.72	0.05	0.63	0.82
		27	0.73	0.04	0.65	0.8
		25	0.68	0.05	0.59	0.78
SLP	→	30	0.75	0.04	0.67	0.83
		29	0.65	0.04	0.56	0.73
		28	0.67	0.04	0.59	0.74
ANX	↔	STR	0.87	0.03	0.81	0.92
ANX	↔	WB	−0.4	0.06	−0.52	−0.28
ANX	↔	SLP	0.67	0.05	0.59	0.76
STR	↔	WB	−0.45	0.06	−0.57	−0.34
STR	↔	SLP	0.75	0.04	0.68	0.83
WB	↔	SLP	−0.4	0.07	−0.53	−0.26

ANX = Anxiety, STR = Stress, WB = Well-being, SLP = Sleep Problems. Est = standardized parameter estimate, SE = standard error. 95% CI = Confidence Intervals, LL = Lower Limit, UL = Upper Limit. One-headed arrows (→) are standardized factor loadings, and double-headed arrows (↔) represent correlations.

#### 3.1.4 Convergent and discriminant validity in short and full version of the LA-MHS

Convergent and discriminant validity were assessed using Average Variance Extracted (AVE) and the Fornell-Larcker criterion (Fornell and Larcker, 1981). As shown in Table 4, for the full version of the LA-MHS, convergent validity was supported for ANX and STR (AVE > 0.50); however, WB (AVE = 0.38) and SLP (AVE = 0.48) fell below the preferred threshold, suggesting higher levels of unique item variance. For the short version (LA-MHS-12), convergent validity was met for ANX, STR, and WB (AVE > 0.50), although SLP fell below the suggested threshold (AVE = 0.48). Regarding discriminant validity, the Fornell-Larcker criterion was not fully met. Specifically, for the full version of the LA-MHS, the factor correlations between STR and ANX (r = 0.84) and STR and SLP (r = 0.80) exceeded the square root of their respective AVEs (Table 4). In the LA-MHS-12, the factor correlation between STR and ANX (r = 0.87) also exceeded the square root of its AVE. These results indicate a high degree of construct overlap, especially between ANX and STR in both versions, which is theoretically expected given the shared affective nature of psychological distress and its known impact on sleep quality (Lund et al., 2010; Daviu et al., 2019).

### 3.2 Phase 2 data-driven approach: cross-validation

#### 3.2.1 Exploratory factor analysis in training sample

Parallel analysis conducted on half of the available sample (training sample; n = 154) suggested a three-factor solution. An exploratory factor analysis (EFA) with oblique rotation (oblimin), specifying the extraction of three factors, yielded a structure broadly consistent with the theoretically proposed dimensions of Anxiety, Stress, and Well-being (Table 6). Factor 1 comprised items 11 to 20, which were originally designed to assess Stress. Notably, this factor also included the three items initially intended to measure Sleep Problems (items 28–30). This pattern suggests that, in this sample, sleep-related symptoms cluster with stress-related indicators, supporting the interpretation of this factor as a Stress (STR) dimension that encompasses sleep disturbances frequently co-occurring with elevated stress levels. Factor loadings for STR ranged from 0.49 to 0.82, with the highest loading observed for Item 11 (“I have felt under pressure most of the time”). Factor 2 corresponded closely to the *a priori* Anxiety (ANX) factor, as it included items 1 to 10, with the exception of Item 4. This item exhibited low (< 0.40) and non-specific loadings across multiple factors and was therefore removed from the subsequent analysis. Loadings for the ANX factor ranged from 0.42 to 0.78, with the highest loading observed for Item 9 (“I have felt that something bad might happen without a clear reason”). Factor 3 aligned with the theoretically proposed Well-being (WB) factor, comprising items 21 to 27, except for Item 24, which showed low (< 0.40) and non-specific loadings was also removed. The highest loading within this factor corresponded to Item 23 (“I have felt satisfied with my personal and work life”).

**Table 6:** Exploratory factor analysis in training sample (*n* = 154).

Item	STR	ANX	WB	h2	u2
1		0.74		0.54	0.46
2		0.78		0.62	0.38
3		0.67		0.64	0.36
4				0.37	0.63
5		0.62		0.55	0.45
6		0.71		0.66	0.34
7		0.74		0.57	0.43
8		0.42		0.54	0.46
9		0.78		0.61	0.39
10		0.43		0.47	0.53
11	0.82			0.65	0.35
12	0.64			0.53	0.47
13	0.76			0.59	0.41
14	0.8			0.72	0.28
15	0.76			0.75	0.25
16	0.64			0.55	0.45
17	0.78			0.48	0.52
18	0.65			0.50	0.50
19	0.72			0.60	0.40
20	0.61			0.51	0.49
21			0.53	0.37	0.63
22			0.65	0.43	0.57
23			0.75	0.63	0.37
24				0.13	0.87
25			0.53	0.36	0.64
26			0.56	0.42	0.58
27			0.71	0.58	0.42
28	0.56			0.37	0.63
29	0.49			0.40	0.60
30	0.58			0.37	0.63

ANX = Anxiety, STR = Stress, WB = Well-being, h2 = communalities, u2 = uniqueness.

#### 3.2.2 CFA and reliability in testing sample: model with three correlated factors

The factor structure identified in the training sample was subsequently evaluated in the independent testing sample (*n* = 154). The CFA specified a model with three correlated factors: ANX (items 1–3 and 5–10), STR-S (items 11–20 and 28–30), and WB (items 21–23 and 25–27). Global fit indices indicated acceptable model fit, χ^2^ (scaled) = 714.28, df = 347, *p* < 0.001, CFI = 0.927, TLI = 0.920, RMSEA = 0.083, SRMR = 0.078. Although RMSEA slightly exceeded conventional thresholds (0.083), this result may be influenced by the use of the WLSMV estimator and the relatively modest sample size in this phase (n = 154), conditions under which RMSEA is known to be biased upward (Shi et al., 2019). Standardized factor loadings were positive and statistically significant, ranging from 0.46 to 0.87 (Table 7). The highest-loading item for the ANX factor was item 6 (“I have felt nervous without an apparent reason”), for STR-S it was item 16 (“I have felt easily irritated or frustrated”), and for WB it was item 27 (“I have felt that my daily actions align with what matters to me”). Interfactor correlations were statistically significant and in the expected directions, with particularly strong associations observed between ANX and STR-S (r = 0.90, SE = 0.02, *p* < 0.001). Internal consistency estimates were excellent for both ANX and STR factors (ANX: *α* = 0.91, *ω* = 0.93; STR-S: α = 0.91, ω = 0.93), and good for WB (α = 0.80, ω = 0.81). Taken together, these findings provide initial empirical support for a three-factor correlated structure—anxiety, stress (including sleep-related problems), and well-being—as well as adequate internal consistency of the corresponding scores.

**Table 7:** Standardized parameters of the confirmatory factor analysis in the testing sample (*n* = 154).

Factor	Parameter	Item/Factor	Est	SE	CI 95%	
					LL	UP
ANX	→	1	0.65	0.05	0.54	0.75
		2	0.76	0.04	0.69	0.84
		3	0.81	0.03	0.75	0.87
		5	0.80	0.03	0.73	0.86
		6	0.87	0.02	0.82	0.92
		7	0.77	0.03	0.71	0.84
		8	0.75	0.04	0.67	0.83
		9	0.80	0.03	0.74	0.86
		10	0.80	0.03	0.73	0.87
STR	→	11	0.79	0.03	0.72	0.86
		12	0.78	0.04	0.71	0.85
		13	0.74	0.04	0.67	0.81
		14	0.74	0.04	0.67	0.82
		15	0.80	0.03	0.74	0.86
		16	0.87	0.02	0.82	0.92
		17	0.68	0.04	0.60	0.77
		18	0.69	0.04	0.61	0.77
		19	0.85	0.02	0.81	0.90
		20	0.72	0.04	0.64	0.81
		28	0.49	0.06	0.37	0.62
		29	0.46	0.06	0.34	0.58
		30	0.65	0.05	0.55	0.75
WB	→	21	0.57	0.08	0.42	0.73
		22	0.59	0.06	0.47	0.70
		23	0.73	0.06	0.61	0.85
		25	0.72	0.06	0.61	0.84
		26	0.64	0.06	0.53	0.76
		27	0.75	0.06	0.64	0.87
ANX	↔	STR	0.90	0.02	0.86	0.93
ANX	↔	WB	−0.36	0.09	−0.53	−0.19
STR	↔	WB	−0.36	0.09	−0.52	−0.19

ANX = Anxiety, STR = Stress, WB = Well-being, Est = standardized parameter estimate, SE = standard error. 95% CI = Confidence Intervals, LL = Lower Limit, UL = Upper Limit. One-headed arrows (→) are standardized factor loadings, and double-headed arrows (↔) represent correlations.

### 3.3 Phase 3: estimation and comparison of competing models

The high interfactor correlations observed in the correlated four-factor model suggested the plausibility of alternative latent structures, particularly a higher-order model and a bifactor model. Using the full sample (*n* = 308), these two competing models were estimated and compared with the original correlated four-factor model specified *a priori* (without residual correlations).

#### 3.3.1 Model fit comparison

In terms of global fit, the baseline correlated four-factor model yielded χ^2^ (scaled) = 1126.83, df = 399, p < 0.001, CFI = 0.923, TLI = 0.916, RMSEA = 0.077, SRMR = 0.067. The higher-order model showed nearly identical fit, χ^2^ (scaled) = 1121.03, df = 401, p < 0.001, CFI = 0.923, TLI = 0.917, RMSEA = 0.076, SRMR = 0.067. In contrast, the bifactor model demonstrated improved fit indices, χ^2^ (scaled) = 836.15, df = 375, p < 0.001, CFI = 0.951, TLI = 0.943, RMSEA = 0.063, SRMR = 0.050. These results indicate that the correlated four-factor and higher-order models provide virtually equivalent fit, whereas the bifactor model shows substantially better global fit according to conventional fit indices. Statistical comparisons using the Likelihood Ratio Test (LRT) further clarified these differences. The comparison between the bifactor and higher-order models indicated that the higher-order model fit the data significantly worse (Δχ^2^(26) = 208.71, *p* < 0.001). In contrast, the comparison between the higher-order model and the baseline correlated four-factor model revealed no statistically significant difference in fit (Δχ^2^(2) = 4.64, *p* = 0.098). Although these comparisons appear to favor the bifactor model, it is well established that bifactor models, due to their greater flexibility and parameterization, are prone to overfitting the data. Therefore, additional indices are recommended to evaluate their substantive interpretability.

#### 3.3.2 Evaluation of the bifactor model

To further assess the viability of the bifactor solution, Explained Common Variance (ECV) was computed. The bifactor model yielded an ECV value of 0.683, which falls below the commonly recommended threshold (ECV > 0.70) for supporting essential unidimensionality. This result suggests that, although the bifactor model provides better statistical fit, the general factor does not account for a sufficiently large proportion of the common variance to justify a unidimensional interpretation of the scale. Accordingly, the original correlated four-factor model specified *a priori* appears to potentially provide a parsimonious representation of the latent structure of the LA-MHS.

## 4 Discussion

The present study aimed to develop and psychometrically evaluate a multidimensional instrument to assess anxiety (ANX), stress (STR), subjective well-being (WB), and sleep problems (SLP) in a sample of Latin American workers. The psychometric evaluation was conducted in three phases. The first phase (theory-driven) examined the factorial structure and reliability of the four factors specified *a priori* during scale development. The second phase (data-driven) employed cross-validation procedures to explore the factor structure in one subset of the sample and evaluate its fit in an independent subset. The third phase involved the estimation and comparison of competing latent structures, specifically higher-order and bifactor models.

Findings from the first phase provided initial support for the reliability and internal structure of the ANX and STR factors. However, they also highlighted the need to refine the content and wording of the WB and SLP dimensions, both in the full version of the LA-MHS (30 items) and, more critically, in the brief version (LA-MHS-12). These results suggest that while distress-related dimensions are well captured, the measurement of protective and sleep-related constructs may require further conceptual and psychometric refinement. The second phase identified a three-factor structure consisting of Anxiety (ANX), a combined Stress and Sleep Problems factor (STR-S), and Well-being (WB). This structure was largely consistent with the theoretically proposed model but revealed some important deviations. Specifically, two items were removed due to inadequate loadings (one from ANX and one from the stress/sleep domain), and the sleep-related items loaded onto the stress factor. This pattern suggests that, in this sample, sleep disturbances may be more closely embedded within the stress response rather than functioning as a fully distinct construct. Although this three-factor solution appears viable, it should be interpreted cautiously pending further validation. The third phase demonstrated that the correlated four-factor model proposed a priori exhibited fit comparable to that of the higher-order model, indicating that the latter does not provide a meaningful improvement in explaining the relationships among dimensions. Although the bifactor model showed superior global fit indices, a more rigorous evaluation using indices specific to bifactor models indicated that the general factor did not account for a sufficient proportion of common variance to support a unidimensional interpretation of the scale. These findings highlight the importance of complementing global fit indices with substantive evaluation when interpreting complex models.

Taken together, the results provide partial support for the originally proposed four-factor structure of the LA-MHS, particularly for the anxiety and stress dimensions. At the same time, they underscore the need for further refinement of the well-being and sleep domains. The findings also support the potential utility of a brief version of the scale, although improvements in the well-being dimension and validation in independent samples are necessary before broader application. Additionally, the data-driven phase suggests that a three-factor structure (ANX, STR-S, WB) may represent a plausible alternative model, although it requires further empirical confirmation. Finally, consistent with the results of the model comparison analyses, more parsimonious factor structures—such as the correlated factors model—are preferable over more complex alternatives like higher-order or bifactor models for representing the latent structure of the LA-MHS.

### 4.1 Interrelations between factors

In the first phase of the psychometric evaluation, interfactor correlations were consistent with the hypothesized pattern: Anxiety (ANX), Stress (STR), and Sleep Problems (SLP) were positively associated, whereas Well-being (WB) was negatively associated with the distress-related dimensions. Among these associations, the strong correlation between Anxiety and Stress merits particular attention. Although conceptually distinct, these constructs share several underlying mechanisms, including sustained physiological activation, cognitive perseveration, and difficulties in emotion regulation. From a transdiagnostic perspective, the magnitude of their association may reflect partially overlapping vulnerability processes rather than redundancy (Harvey, 2008). In this context, it is noteworthy that the strongest factor loadings were observed for items reflecting emotional exhaustion (Stress) and unexplained nervousness (Anxiety), which are widely recognized as core manifestations of psychological distress (Kessler et al., 2002). This pattern is consistent with established theoretical frameworks of occupational mental health. Within Maslach’s burnout model, emotional exhaustion represents the central component of chronic work-related strain (Maslach et al., 1996). Similarly, the Job Demands–Resources model conceptualizes sustained psychological demands as key drivers of emotional fatigue and impaired functioning (Bakker and Demerouti, 2017; Schaufeli and Taris, 2014). The prominence of these indicators in the present model suggests that the LA-MHS may be sensitive to central features of work-related distress, although this interpretation should be considered preliminary.

The consistently high correlations observed between Stress and Anxiety across models (as well as between Stress and Sleep Problems in Phase 1) also raise the possibility of alternative latent structures. In an exploratory manner, two competing models were evaluated. The first was a higher-order model, in which a general factor accounts for the associations among the first-order factors (ANX, STR, WB, and SLP). The second was a bifactor model, in which a general factor explains variance across all items, while orthogonal group factors capture residual variance specific to each domain. In the present sample, neither the higher-order nor the bifactor model provided a clearly more viable representation of the data when considering both statistical evidence and theoretical interpretability. Beyond statistical considerations, these models also imply the presence of a single common underlying cause driving all measured phenomena. While stress, anxiety, and sleep problems may share partially overlapping processes, they do not constitute a unitary construct, nor do their manifestations necessarily arise from a single source.

A simple illustration of this complexity is that stress responses are typically elicited by identifiable external demands, whereas anxiety-related responses may occur in the absence of a clear stimulus (Cheng and McCarthy, 2018). In turn, both stress and anxiety can contribute to sleep disturbances, suggesting directional and potentially reciprocal relationships rather than a single latent cause. Chronic stress disrupts sleep through sustained physiological arousal, while sleep impairment compromises emotional and cognitive regulation, increasing vulnerability to anxiety and stress. In this sense, sleep difficulties function not only as correlates of distress but also as amplifying mechanisms within broader vulnerability networks, as consistently reported in sleep and psychopathology research (Baglioni et al., 2010; Baglioni et al., 2011; Alvaro et al., 2013). For this reason, some authors have argued that psychological phenomena may be better conceptualized using complex systems approaches (e.g., network models) rather than traditional common-cause models such as factor analysis (Robinaugh et al., 2019). However, examining such network structures for the constructs assessed by the LA-MHS falls beyond the scope of the present study.

Finally, the negative association between Well-being and the distress-related dimensions provides empirical support for the two-continua model of mental health. The findings reinforce the notion that mental health cannot be conceptualized solely as the absence of symptoms; rather, well-being represents a related yet partially independent dimension reflecting positive functioning and psychological resources. The inclusion of well-being is consistent with the intended integrative nature of the LA-MHS by trying to capture both vulnerability and protective factors within a single test.

### 4.2 Brief version: LA-MHS-12

The development of the brief version (LA-MHS-12) represents a preliminary effort to create a more parsimonious and time-efficient instrument. The results provide initial support for its internal structure, particularly for the Anxiety and Stress dimensions, which showed relatively stable and interpretable patterns. However, by design, the brief version entails reduced content coverage, which may limit its ability to fully capture the breadth and complexity of the constructs, especially for Well-being. Although the LA-MHS-12 demonstrated acceptable model fit in the present sample, this finding should be interpreted with caution, as the same dataset was used for both item selection and model evaluation. As a result, model fit and parameter estimates may be optimistically biased.

Accordingly, the LA-MHS-12 should be considered a provisional abbreviated version, and further research is required before it can be recommended for applied use. In particular, future studies should: (1) Empirically evaluate the trade-off between efficiency and content validity, examining whether reductions in administration time and burden justify the potential loss of construct coverage. (2) Establish reliability and internal consistency for each factor in independent samples. (3) Confirm the internal structure and examine associations with external variables to strengthen the validity of score interpretations, and (4) estimate the degree of shared variance between the full and brief versions, to determine the extent to which the short form adequately represents the original instrument (e.g., Smith et al., 2000; Marsh et al., 1998). Overall, while the LA-MHS-12 shows promise as a brief screening tool, its use should remain cautious and exploratory until further psychometric evidence is established.

### 4.3 Well-being and sleep problems factors and *post hoc* modifications

The Well-being and Sleep factors emerged as the most psychometrically vulnerable components of the instrument. In particular, the need to introduce post hoc residual correlations among several Well-being items suggests potential limitations in item content and wording, such as redundancy or insufficient conceptual differentiation. Residual correlations were specified for four item pairs (24–23, 24–26, 24–21, and 23–22) to account for local dependence. These correlations were theoretically justified by a combination of semantic overlap—where items share closely related emotional descriptors (e.g., “joy” and “satisfaction”)—and functional contingency, in which specific experiences (e.g., goal attainment or social valuation) may act as proximal determinants of broader well-being, generating shared variance not fully captured by the latent factor.

More specifically, Item 24 (“joy”) reflects a high-arousal positive affective state that overlaps with multiple aspects of well-being. Its association with Item 23 (“satisfaction”) may reflect semantic redundancy, as feeling joyful and satisfied can be experienced similarly, leading to highly correlated responses. Its correlation with Item 26 (“valued by others”) may reflect a functional relationship, as feelings of joy are often a consequence of perceived social recognition. Likewise, the association with Item 21 (“peace”) may indicate that joy and inner peace represent different expressions of a broader positive affective experience, with peace reflecting a lower-arousal manifestation. Finally, the correlation between Item 23 (“satisfaction”) and Item 22 (“motivation/goals”) may reflect a functional dependency consistent with goal-setting frameworks, in which satisfaction arises as a consequence of goal attainment. Taken together, these patterns suggest that the Well-being factor, as currently operationalized, may represent a heterogeneous construct that is not fully captured by a single latent dimension. This points to the need for refinement in both item wording and content coverage, and potentially for the exploration of subdimensions of well-being (e.g., goal-related well-being, social validation, or productivity-related well-being), which may require additional items for adequate measurement.

Similarly, the relatively low reliability observed for the Sleep Problems factor, along with its tendency to load onto the Stress dimension in the exploratory analyses, indicates that this domain may also require substantial revision, either in terms of item formulation or conceptual definition. One possible interpretation, suggested by the cross-validation results in Phase 2, is that sleep problems may be more appropriately conceptualized as consequences of stress dysregulation or poor anxiety management, rather than as a fully independent construct. However, this interpretation remains tentative and requires further empirical investigation.

### 4.4 Cultural relevance and organizational applicability

A distinctive contribution of the LA-MHS lies in its cultural grounding. Many widely used instruments assessing anxiety, stress, and sleep were developed and validated primarily in English-speaking contexts. Although these instruments have demonstrated strong psychometric properties, cross-cultural adaptation can present challenges related to semantic equivalence and contextual relevance. The LA-MHS was developed with explicit consideration of linguistic clarity and cultural appropriateness for Latin American working populations. This contextualization enhances its potential applicability in organizational settings across the region, where working conditions, organizational cultures, and expressions of psychological distress may differ from those observed in other sociocultural contexts.

### 4.5 Limitations

Several limitations should be considered. First, the cross-sectional design precludes conclusions regarding temporal stability, causal relations among dimensions, or predictive validity. Longitudinal research is needed to evaluate test–retest reliability, sensitivity to change, and the scale’s capacity to detect improvements following intervention. Second, the sample was obtained through non-probabilistic convenience sampling strategy and was characterized by an overrepresentation of women, younger individuals and participants from specific occupational sectors. Additionally, recruitment through LinkedIn, referral networks, and participating organizations may have introduced selection biases, such as the inclusion of individuals with greater access to digital platforms or higher interest in mental health topics. These characteristics may limit the generalizability of the findings, particularly to other occupational sectors, age groups, or more diverse working populations. Accordingly, the results should be interpreted as preliminary and may not fully represent the broader workforce. Future research should aim to replicate these findings in more diverse and representative samples. Third, although internal structure was supported through CFA, additional sources of validity evidence remain a key limitation of the present study and they need to be further investigated. These include measurement invariance across gender, age, and country; convergent validity with established instruments; and external criterion validity with respect to organizational outcomes such as absenteeism, performance, or turnover intentions.

## 5 Conclusion

The LA-MHS demonstrates promising initial psychometric properties, especially in assessing anxiety and stress. Nevertheless, further work is required to refine the well-being and sleep problems dimensions, evaluate alternative structural models (e.g., three correlated factors) using a fully independent sample, and establish external criterion validity. At its current stage, the instrument should be considered a developing measure, with potential for application pending additional validation.

## Data Availability

The raw data supporting the conclusions of this article will be made available by the authors, without undue reservation.
